# Expectations about the effectiveness of pain‐ and itch‐relieving medication administered via different routes

**DOI:** 10.1002/ejp.1163

**Published:** 2017-12-20

**Authors:** K.J. Peerdeman, J. Tekampe, A.I.M. van Laarhoven, H. van Middendorp, R.C.A. Rippe, M.L. Peters, A.W.M. Evers

**Affiliations:** ^1^ Health, Medical and Neuropsychology Unit Leiden University The Netherlands; ^2^ Leiden Institute for Brain and Cognition Leiden University The Netherlands; ^3^ Department of Psychiatry Leiden University Medical Center The Netherlands; ^4^ Centre for Child and Family Studies Leiden University The Netherlands; ^5^ Department of Clinical Psychological Science Maastricht University The Netherlands

## Abstract

**Background:**

Placebo effects on pain have been found to vary in size for different routes of medication administration (e.g. oral vs. injection). This has important implications for both clinical research and practice. To enhance our understanding of these differential placebo effects, research on the underlying expectations about multiple routes and symptoms other than pain is vital.

**Methods:**

A cross‐sectional, Internet‐based survey was conducted in a representative sample of the Dutch population (*n *=* *508). Respondents rated the expected effectiveness of pain‐ and itch‐relieving medication in six forms, representing oral, injection and topical routes of administration.

**Results:**

Injected medication was expected to be most effective for relieving pain, and topical medication for relieving itch. Furthermore, exploratory analyses showed that injections were expected to have the most rapid onset and long‐lasting effects, and to be most frightening and expensive, while topical medication was expected to be the safest and the easiest to use, and oral medication was expected to have the most side effects. Higher expected effectiveness was moderately associated with expectations of more rapid onset and long‐lasting effects, and better safety and ease of use. Associations of expected effectiveness with respondent characteristics (e.g. medication use and personality characteristics) were statistically small or nonsignificant.

**Conclusions:**

Expected effectiveness of medication differed depending on route of administration and targeted symptom. These findings have important implications for the design and interpretation of clinical trials and suggest that medication effects might be enhanced by prescribing medicine via the route that patients expect to be most effective for their complaint.

**Significance:**

Differences in the expected effectiveness of medication depend on the route of administration (oral, injection, topical) and targeted symptom (pain, itch). These findings have important implications for clinical practice and the design and interpretation of clinical trials.

## 1 Introduction

Placebos have repeatedly been found to relieve pain and other symptoms, presumably through expectancies (Kirsch, 1997; Benedetti, 2014; Peerdeman et al., 2016a). Not all placebos affect pain equally. An important treatment characteristic that has been associated with differential placebo effects on pain is the route of medication administration. It is frequently suggested that more invasive routes of medication administration (such as injections) lead to enhanced placebo effects (Lasagna, 1955; Kaptchuk et al., 2000; Schwartz et al., 2000). Indeed, placebo injections have been found to be more effective for relieving pain than oral placebos (de Craen et al., 2000; Zhang et al., 2008; Bannuru et al., 2015; Peerdeman et al., 2016a). However, many research findings, looking also into other routes, are mixed regarding the possible enhanced effectiveness of more invasive routes for relieving pain (Macedo et al., 2006; Meissner et al., 2013; Bannuru et al., 2015; Fassler et al., 2015), while one study did not show substantial differences between different routes at all (Schwartz et al., 2000). Since differential placebo effects have important implications for clinical trials and clinical practice, further research into the underlying expectations about the effectiveness of medication administered via different routes is required.

For further research, several factors should be considered. First, most previous research compared the placebo control conditions of separate clinical trials, while direct comparisons between multiple routes of medication administration are relatively scarce. Second, research is generally limited to pain, while research into multiple symptoms is vital to examine whether differential placebo effects of different routes may depend on the targeted symptom. In this regard, itch is of particular interest. Like pain, itch imposes a heavy burden on many patients (Weisshaar and Dalgard, 2009; Matterne et al., 2011), and the underlying mechanisms of pain and itch overlap considerably (Ständer and Schmelz, 2006; Schmelz, 2015). Only one meta‐analysis has assessed differential placebo effects on itch, suggesting that oral and injected placebos did not differ (van Laarhoven et al., 2015). Comparisons with topical routes, which are most frequently used for itch, could however not be made in this analysis. In addition, our understanding of differential placebo effects can be improved by looking at expectations about other characteristics, such as side effects and cost (Waber et al., 2008; Berna et al., 2017), which have previously been found to affect placebo effects. Also, possible correlates of the expectations about the effectiveness of medication, including these other characteristics of the routes and respondent characteristics (e.g. frequency of medication use and personality characteristics (Colloca and Miller, 2011; Horing et al., 2014)), are rarely explored.

In this cross‐sectional study, we used a survey to directly compare expectations about medication administered via three common routes for relieving pain and itch in a large sample representative of the Dutch population. Our primary aim was to assess differences between the expected effectiveness of medication administered via oral (tablet, capsule), injection (syringe, infusion) and topical (cream, gel) routes for relieving pain and itch. In addition, we explored expectations about multiple other characteristics of the routes (i.e. side effects, long‐lasting effect, rapid onset, safety, being frightening, cost and ease of use), as well as possible correlates of the expected effectiveness (i.e. expectations about the aforementioned characteristics of the routes, and the following respondent characteristics: demographics, health, frequency of medication use, medication attitude and personality characteristics).

## 2 Methods

### 2.1 Respondents

The sample consisted of adults (≥18 years) who were fluent in the Dutch language. Respondents were recruited via online research panels; Qualtrics (Provo, UT, USA) panel members from the Dutch population were invited via e‐mail to complete the online survey in return for incentives or cash honorarium, according to the standard procedures of Qualtrics. To obtain a sample that was representative of the adult Dutch population in terms of age, sex and province of residence (Centraal Bureau voor de Statistiek, 2015), the data of respondents who were over quota were not analysed.

### 2.2 Procedure

The study protocol was approved by the institute's ethics committee (*Commissie Ethiek Psychologie*, PREC15‐0828_33). The study was a cross‐sectional, Internet‐based survey. After providing informed consent, upon receiving information about the study purpose and procedures, respondents filled out a series of questionnaires via the secured online system Qualtrics (Provo, UT, USA). Median completion time was 19 min. Data collection took place in autumn 2015.

### 2.3 Questionnaires

#### 2.3.1 Expectations about medication

A questionnaire developed specifically for this study was used to measure respondents’ expectations about six different forms of medication administration, representing three common routes of administration, specifically oral (i.e. tablet, capsule), injection (i.e. syringe, infusion) and topical (i.e. cream, gel) routes. See Supporting Information Appendix S1 for an English version of the questionnaire. This questionnaire evolved from a pilot study conducted in a sample of 100 volunteers (mostly young female university students), which provided preliminary indications that expected effectiveness of medication depends on the route of administration and targeted symptom. Based on the pilot, the questionnaire was optimized for the current research questions (e.g. rephrasing questions, focus on specific routes, symptoms and characteristics). First, a brief description of each of the forms of administration was shown along with a photograph on which the form was presented in a standardized manner (see Supporting Information Appendix S1). Subsequently, respondents rated the expected effectiveness of pain‐ and itch‐relieving medication administered in the different forms (“How effective do you think pain‐relieving/itch‐relieving medications are when they are used in the following forms?”) on a horizontal visual analogue scale (VAS) ranging from *not effective at all* (0) to *very much effective* (100). Next, respondents rated to what extent they expected seven other characteristics to be applicable to each of the forms of administration, irrespective of the targeted symptom, specifically: (1) side effects, (2) long‐lasting effect, (3) rapid onset, (4) safe, (5) frightening, (6) expensive, (7) easy to use. These items were rated on a horizontal VAS ranging from *not at all applicable* (0) to *very much applicable* (100). While the aforementioned subparts of the questionnaire (i.e. expected effectiveness and expected other characteristics) were always presented in the same order, the presentation of the forms of medication administration, symptoms and other characteristics within these subparts was automatically randomized.

#### 2.3.2 Demographics

Respondents reported several demographic characteristics, including age, sex, province of residence, educational level, nationality, mother tongue, fluency in Dutch language, religious or ideological affiliation and marital status.

#### 2.3.3 Health

To assess health, respondents answered questions about being in treatment for long‐lasting (≥1 month) medical or psychological complaints or diseases (e.g. diabetes, pain, high blood pressure or depression; dichotomous scale), presence of chronic pain (≥3 months) or itch (≥6 weeks) at present or in the past (dichotomous scale) and intensity of current pain and itch (0–100 VAS). The Short Form‐12 (SF‐12) (Mols et al., 2009) was used to measure health status (12 items, various Likert scales). Scores on the physical component summary and the mental component summary of the SF‐12 were calculated using item response theory (Mols et al., 2009), with higher scores indicating a better physical or mental health status, respectively.

#### 2.3.4 Frequency of medication use

To assess medication use, respondents reported how often they used pain‐ and itch‐relieving medication in each form of administration throughout their lives (7‐point Likert scale, higher scores indicate more frequent use).

#### 2.3.5 Medication attitude

To measure general beliefs about the harmfulness of medication and doctor's over‐prescription of medication, the general harm and overuse scales of the Beliefs about Medication Questionnaire (BMQ) (Horne et al., 1999) were used (2 × 4 items, 5‐point Likert scale). The total score of each scale ranges from 4 to 20, with higher scores indicating more negative beliefs. Cronbach's alpha was 0.73 for the harm scale and 0.78 for the overuse scale in this study. Respondents also reported whether they were employed in health care at any time point and, if so, whether they prescribed medication to patients (dichotomous scales).

#### 2.3.6 Personality characteristics

To measure dispositional optimism, the revised Life Orientation Test (LOT‐R) (Scheier et al., 1994) was used (three positive, three negative and four filler items, 5‐point Likert scale). The total score ranges from 0 to 24, with higher scores indicating higher optimism. Cronbach's alpha was 0.76 in this study. To measure neuroticism, the neuroticism scale of the revised short version of the Eysenck Personality Questionnaire (EPQ‐RSS) (Sanderman et al., 1995) was used (12 items, dichotomous scale). The total score ranges from 0 to 12, with higher scores indicating more neuroticism. Cronbach's alpha was 0.88 in this study.

### 2.4 Response quality

To assess whether respondents were paying attention to the questions, two control items were included (after around 1/3 and 2/3 of the survey) (Desimone et al., 2015) that instructed respondents to answer on the lowest or highest end of a 0–100 VAS, respectively. Answers deviating more than 10 points from the required answer were considered incorrect. The survey ended with two questions to assess how well respondents understood and read the questions (4‐point Likert scale) to filter out respondents who did not understand or read many or all questions well. Respondents were also given the opportunity to report questions and remarks, and survey completion time was recorded. Using *forced response* validation, participants were required to answer all questions to prevent missing data.

### 2.5 Statistical analyses

The six different forms of medication administration were grouped into three categories – indicating the oral (i.e. tablet and capsule), injection (i.e. syringe and infusion) and topical (i.e. cream and gel) routes of medication administration – by averaging the values of the two forms within each category. Confirmatory principal component analysis with oblimin rotation confirmed this three‐factor structure of the expected effectiveness for both relieving pain and itch, separately (see Supporting Information Tables S1 and S2).

For the primary research question, regarding the expected effectiveness of medication administered via the different routes for relieving pain and itch, a 3 × 2 repeated measures analysis of variance (RM‐ANOVA) was used. Within‐subjects independent variables were (1) route of medication administration (oral, injection or topical) and (2) symptom (pain or itch), and the dependent variable was expected effectiveness. First, the interaction effect of route‐by‐symptom was inspected. If the interaction was significant, the main effects of route on expected effectiveness were analysed with separate RM‐ANOVAs for pain and itch. In case no interaction of route‐by‐symptom was observed, the main effect of route was examined irrespective of symptom. If a significant main effect of route was observed, pairwise comparisons between the different routes of administration were examined.

Expectations about other characteristics of the routes of medication administration (e.g. side effects), which were assessed irrespective of symptom, were explored using a separate RM‐ANOVA for each of the characteristics. For each analysis, the independent variable was the route (oral, injection or topical), and the dependent variable was the expectation about the characteristic. If a significant main effect of route was observed, pairwise comparisons between the different routes were examined.

Furthermore, we explored possible correlates of expected effectiveness of medication. For continuous variables, correlation analyses were used to explore the association of expected effectiveness of medication overall (i.e. mean value across routes and symptoms) with expectations about the other characteristics of the routes and with respondent characteristics. For categorical variables, univariate ANOVAs were used with the characteristics as between‐subjects independent variable and expected effectiveness of medication overall as dependent variable.

All data were analysed using SPSS Statistics version 23 (IBM Corporation, Armonk, NY, USA), with a two‐tailed significance level of α = 0.05. For the primary analyses, the inheritance procedure was used to correct for multiple testing (3/5α for main effects, α/4 for contrasts (Goeman and Finos, 2012)). For the additional analyses, the *p* values were not corrected given the exploratory nature of these analyses. Because of the large sample size and number of analyses, we focused on effect sizes rather than on *p* values. For (RM) ANOVAs, generalized eta squared ($\eta_{G}^{2}$) was calculated, with 0.01, 0.06 and 0.14 indicating small, medium and large effects, respectively (Lakens, 2013). For correlation analyses, Pearson's *r* values of 0.10, 0.30 and 0.50 were interpreted as indicating small, medium and large correlations, respectively (Cohen, 1988). In case the assumptions of the RM‐ANOVAs for the primary analyses were violated, sensitivity analyses were conducted using (1) transformed data and/or (2) winsorized data, that is where the effect of outliers (absolute *z* score >3.29) is reduced by replacing the raw score with the most extreme raw score that was not an outlier, plus/minus 1 for each consecutive outlier. The results of these sensitivity analyses yielded the same conclusions as the uncorrected analyses. For all RM‐ANOVAs in which variables with more than two levels were compared, violations of the assumption of sphericity were corrected using the Greenhouse–Geisser (if ε < 0.75) or Huynh‐Feldt (if ε > 0.75) procedures.

## 3 Results

### 3.1 Respondents

In total 904 respondents reacted to the invitation to participate in the study. Of these, 112 respondents did not actually begin participation, and 234 respondents did not complete the survey and/or answered one or both of the control questions incorrectly (i.e. deviation of more than 10 points from the required answer). Two respondents were not fluent in the Dutch language. Another 40 respondents were over quota (in terms of sex, age or province of residence). Five respondents completed the survey very fast (in less than 1/3 of the median time, i.e. <6.4 min), causing uncertainty about the reliability of the data, and three respondents indicated not having understood or read many or all questions well. After excluding the data of all these respondents, the complete data of 508 respondents were available for analyses. Demographics, health, frequency of medication use, medication attitude and personality characteristics of the final sample are reported in Table 1.

**Table 1:** Demographics, health, frequency of medication use, medication attitude and personality characteristics of the final sample (*n *=* *508)

	Mean/*n*	*±SD*/%
Demographics		
Age *(range 18–75)*	47.0	(±16.1)
Sex *(% men)*	247	(48.6%)
Educational level		
Primary	6	(1.2%)
Secondary	304	(59.8%)
Tertiary	198	(39.0%)
Nationality		
Dutch	497	(97.8%)
Other	6	(1.2%)
Multiple	5	(1.0%)
Religious or ideological affiliation		
None	300	(59.1%)
Christian	178	(35.0%)
Other	30	(5.9%)
Marital status		
Single	175	(34.4%)
In relationship	333	(65.6%)
Health		
Currently in treatment for long‐lasting medical or psychological complaints or diseases	218	(42.9%)
Chronic pain past	148	(29.1%)
Chronic itch past	66	(13.0%)
Chronic pain present	140	(27.6%)
Chronic itch present	51	(10.0%)
Current pain intensity *(0–100 VAS)*	25.9	(±30.4)
Current itch intensity *(0–100 VAS)*	12.0	(±21.7)
Physical health status (SF‐12)	47.9	(±11.5)
Mental health status (SF‐12)	46.3	(±12.1)
Frequency of medication use		
Frequency of pain‐relieving medication use *(1‐7 Likert scale)*	2.1	(±0.9)
Frequency of itch‐relieving medication use *(1‐7 Likert scale)*	1.3	(±0.5)
Medication attitude		
Beliefs about medication – general harm (BMQ) *(theoretical range 4–20)*	10.6	(±2.7)
Beliefs about medication – general overuse (BMQ) *(theoretical range 4–20)*	12.5	(±3.0)
Health care employee in past or present	75	(14.8%)
If health care employee: prescribed medication in past or present	38	(7.5%)
Personality characteristics		
Optimism (LOT‐R) *(theoretical range 0–24)*	13.9	(±3.8)
Neuroticism (EPQ‐RSS) *(theoretical range 0–12)*	3.8	(±3.6)

### 3.2 Expected effectiveness

**Figure 1:**
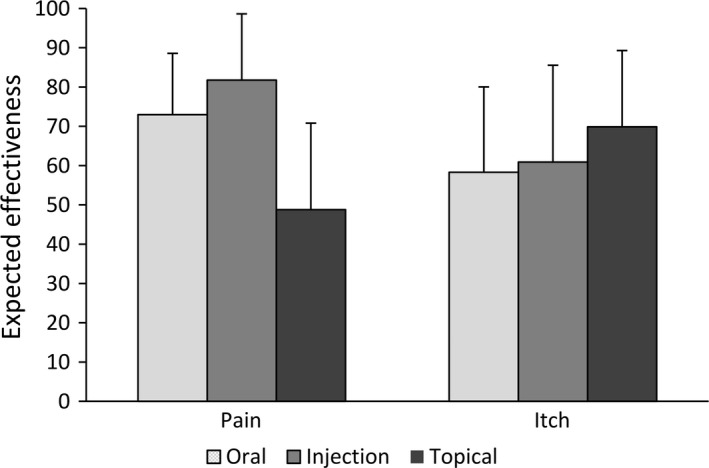
Expected effectiveness of pain‐ and itch‐relieving medication administered via the three routes as rated on a visual analogue scale ranging from *not effective at all* (0) to *very much effective* (100) (mean, error bars indicate standard deviation).

The expected effectiveness of pain‐ and itch‐relieving medication administered via each of the three routes is depicted in Fig. 1 (see Supporting Information Table S3 for the exact values). The RM‐ANOVA showed a large interaction effect of route‐by‐symptom on expected effectiveness (*F* (1.41, 714.22) = 448.99, *p *<* *0.001, $\eta_{G}^{2}$ = 0.24). Subsequent ANOVAs showed a large main effect of route for pain (*F* (1.66, 839.37) = 628.29, *p *<* *0.001, $\eta_{G}^{2}$ = 0.47) and a medium main effect of route for itch (*F* (1.50, 761.86) = 50.12, *p *<* *0.001, $\eta_{G}^{2}$ = 0.07). Pairwise comparisons indicated medium and large differences for pain; injected medication was expected to be more effective than oral medication (*F* (1, 507)* *=* *148.61, *p *<* *0.001, $\eta_{G}^{2}$ **= 0.11) and topical medication (*F* (1, 507) = 875.34, *p *<* *0.001, $\eta_{G}^{2}$ **= 0.50), and oral medication was expected to be more effective than topical medication (*F* (1, 507) = 572.39, *p *<* *0.001, $\eta_{G}^{2}$ **= 0.37). For itch, effect sizes indicated small and medium differences between the routes; topical medication was expected to be more effective than injected medication (*F* (1, 507) = 38.58*, p *<* *0.001*,* $\eta_{G}^{2}$ **= 0.05) and oral medication (*F* (1, 507) = 80.28, *p *<* *0.001, $\eta_{G}^{2}$ = 0.10), and injected medication was expected to be more effective than oral medication (*F* (1, 507) = 10.25, *p *=* *0.006, $\eta_{G}^{2}$ = 0.01).

### 3.3 Other expected characteristics of the routes of administration

**Figure 2:**
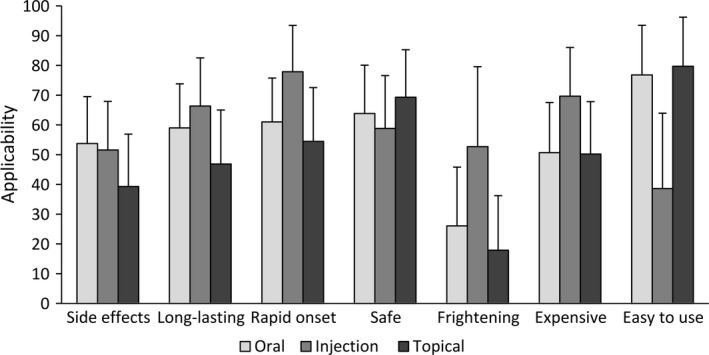
Expectations about other characteristics of the three routes of medication administration (irrespective of the targeted symptom) as rated on a visual analogue scale ranging from *not at all applicable* (0) to *very much applicable* (100) (mean, error bars indicate standard deviation).

Expectations about the other characteristics of the routes of administration (side effects, long‐lasting, rapid onset, safe, frightening, expensive, easy to use) are depicted in Fig. 2 (see Supporting Information Table S3 for the exact values). A significant medium or large main effect of the three routes was observed for all seven characteristics (all *p *<* *0.001, $\eta_{G}^{2}$ = 0.10–0.55). Pairwise comparisons further showed significant differences between all routes for all characteristics (all *p *≤* *0.003), varying in size ($\eta_{G}^{2}$ = 0.01–0.55), with one exception; oral and topical medication did not significantly differ in expected cost (*p *=* *0.53, $\eta_{G}^{2}$ < 0.01). Test‐statistics for all pairwise comparisons are reported in Supporting Information Table S4.

### 3.4 Correlates of expected effectiveness

The test‐statistics of all analyses testing associations of the overall expected effectiveness (irrespective of route and symptom) with other route and respondent characteristics are reported in Table 2 (see Supporting Information Table S5 for associations per route and symptom). A higher expected effectiveness was moderately (*r *≥* *0.30) associated with expectations of medication having more long‐lasting effects, a more rapid onset and being more safe and easy to use (all *p *<* *0.001). Statistically significant but small associations (*r *≥* *0.10 or $\eta_{G}^{2}$ ≥ 0.01) were observed between higher expected effectiveness and expectations of medication being less frightening, having experienced chronic pain in the past, more frequent use of itch‐relieving medication, less negative general beliefs about harm and overuse of medication, and having been or being employed in health care (all *p *<* *0.05). Associations with all other variables did not reach statistical significance (see Table 2).

**Table 2:** Associations between overall expected effectiveness of medication with other route and respondent characteristics

	a	Expected effectiveness^b^
Other expected characteristics of the routes^c^		
Side effects	*r*	−0.06
Long‐lasting effect	*r*	**0.44** ***
Rapid onset	*r*	**0.49** ***
Safe	*r*	**0.42** ***
Frightening	*r*	−0.20***
Expensive	*r*	0.05
Easy to use	*r*	**0.31** ***
Demographics		
Age	*r*	0.04
Sex	$\eta_{G}^{2}$	<0.01
Educational level	$\eta_{G}^{2}$	<0.01
Religious or ideological affiliation	$\eta_{G}^{2}$	<0.01
Marital status	$\eta_{G}^{2}$	<0.01
Health		
Currently in treatment for long‐lasting medical or psychological complaints or diseases	$\eta_{G}^{2}$	<0.01
Chronic pain past	$\eta_{G}^{2}$	0.01*
Chronic itch past	$\eta_{G}^{2}$	<0.01
Chronic pain present	$\eta_{G}^{2}$	0.01
Chronic itch present	$\eta_{G}^{2}$	<0.01
Current pain intensity	*r*	0.05
Current itch intensity	*r*	−0.01
Physical health status (SF‐12)	*r*	−0.07
Mental health status (SF‐12)	*r*	−0.03
Frequency of medication use		
Frequency of pain‐relieving medication use	*r*	0.09
Frequency of itch‐relieving medication use	*r*	0.11*
Medication attitude		
Beliefs about medication – general harm (BMQ)	*r*	−0.11*
Beliefs about medication – general overuse (BMQ)	*r*	−0.16***
Health care employee (past or present)	$\eta_{G}^{2}$	0.02**
If health care employee (*n *=* *76): prescribed medication (past or present)	$\eta_{G}^{2}$	0.05
Personality characteristics		
Optimism (LOT‐R)	*r*	0.08
Neuroticism (EPQ‐RSS)	*r*	−0.01

Medium and large effect sizes are printed in bold.

*p < 0.05, **p < 0.01, ***p < 0.001; *p* values are unadjusted.

a *r *= Pearson correlation coefficient (for continuous variables); $\eta_{G}^{2}$  = generalized eta squared (for categorical variables).

b The overall expected effectiveness is calculated across the different routes of medication administration and symptoms.

c The overall expected characteristics are calculated across the different routes of medication administration.

## 4 Discussion

The current study set out to gain a better understanding of differential placebo effects by studying underlying expectations about the effectiveness of medication administered via different routes for relieving both pain and itch. The survey, in a large and representative sample of the Dutch population, showed for the first time that the expected effectiveness of medication depended not only on the route of medication administration (oral, injection or topical), but also on the targeted symptom (pain or itch). Specifically, while pain‐relieving medication was expected to be most effective when administered via injection (and least effective when administered topically), itch‐relieving medication was expected to be most effective when administered topically (and least effective when administered orally).

Additional exploratory analyses showed that, irrespective of pain or itch symptoms, expectations about characteristics other than effectiveness also differed between the routes. Injections were expected to have the most rapid onset and long‐lasting effects, and to be most frightening and expensive, while topical medication was expected to be the safest and the easiest to use, and oral medication was expected to have the most side effects. An exploration of the correlates of expected effectiveness of medication indicated that a higher expected effectiveness was moderately associated with expectations of medication having more long‐lasting effects, a more rapid onset and being more safe and easy to use. Expected effectiveness was not or only weakly associated with other expected characteristics of the routes (i.e. side effects, frightening, expensive) and the measured respondent characteristics (i.e. demographics, health, frequency of medication use, medication attitude and personality characteristics).

The finding that expectations about the effectiveness of medication differed for different routes of administration is in line with previous research demonstrating differential placebo effects on pain for different routes of medication administration. However, the common belief that more invasive routes (such as injections) are more effective (Lasagna, 1955; Kaptchuk et al., 2000; Schwartz et al., 2000) is challenged by the finding that the expected effectiveness of medication administered via different routes depended on the targeted symptom. Although injections were indeed expected to be most effective for relieving pain, injections were second to topically administered medication for relieving itch. Also, the finding that oral medication was expected to be more effective for relieving pain than topical medication could be interpreted as contradicting this idea, as the topical route is often believed to be more complex than the oral route (e.g. Fassler et al., 2015). Moreover, previous research into pain relief also does not consistently support the idea of enhanced placebo effects for more invasive routes (de Craen et al., 2000; Schwartz et al., 2000; Macedo et al., 2006; Zhang et al., 2008; Meissner et al., 2013; Bannuru et al., 2015; Fassler et al., 2015; Peerdeman et al., 2016a). In addition, we found associations of a higher expected effectiveness with better expected safety and ease of use, but no substantial associations with side effects, being frightening and cost, which also does not support the importance of invasiveness. Especially, the lack of an association with cost is surprising, as previous studies indicated larger placebo effects with expensive versus cheap placebos (Waber et al., 2008; Espay et al., 2015). In sum, invasiveness cannot fully explain differential placebo effects for different routes.

A second explanatory factor for differential expectancies and placebo effects may be previous experiences, as learning accounts of placebo effects suggest they shape expectancies (Colloca and Miller, 2011; Peerdeman et al., 2016b). However, we found no or only small associations of expected effectiveness with frequency of medication use, and with the presence and history of chronic pain and itch. Third, people might also expect medication to be most effective when administered via the most common route. Itch‐relieving medications are indeed most commonly administered topically. However, pain‐relieving medications are most commonly administered orally, rather than via injections. Fourth, the location of symptoms might play a role. Because itch is typically located on the skin, a topical medication seems an obvious choice, and since pain can occur at almost any location in the body, one might expect routes with systemic effects (injections or oral medications) to be more effective for relieving pain. Last, respondent characteristics, particularly personality characteristics that pertain to expectancies (i.e. optimism and neuroticism), have frequently been considered as possible moderators of placebo effects and several studies support this (Horing et al., 2014; Weimer et al., 2015). However, current associations of expected effectiveness with the measured demographics, health, medication attitude and personality characteristics were statistically small or nonsignificant. In sum, multiple factors together, not just invasiveness, appear to underlie differential expectations about effectiveness of medication administered via different routes.

Several limitations of our study need to be acknowledged. First, we did not specify the nature of pain and itch (e.g. duration, location, intensity), nor a specific medication (e.g. over‐the‐counter vs. prescription drug). This allowed us to draw general inferences, but expectations about the different routes might also depend on these specifics. Second, although the current study design allowed us to measure numerous forms of medication administration and possible correlates of expected effectiveness, our assessments are by no means complete. Comparisons with other forms of administration (e.g. rectal) and other types of treatment (e.g. surgery), and associations with other respondent characteristics (e.g. generalized self‐efficacy, genetic variations) might be considered for future research. Also, we did not ask respondents about the quality of their previous experiences with pain‐ and itch‐relieving medication, for example whether they had experienced successful pain or itch relief, but this may significantly influence respondents’ expectancies and should be considered in future research. Third, we consider it a strength of our study that we used a large sample representative of the Dutch population in terms of age, sex and province of residence. Nonetheless, it should be noted that the sample is limited to people who registered to commercial online research panels and our findings may not fully generalize to the whole population or specific patient samples.

The current finding that the expected effectiveness of medication depends on the route of medication administration and the targeted symptom has important implications for clinical research and practice, as patients’ expectations are important predictors of placebo effects and hence treatment outcome (Peerdeman et al., 2016b). It challenges the classic interpretation of placebo‐controlled trials, as their results do not only depend on responses to the active medication but also depend on responses to the placebo. As illustrated by the efficacy paradox (Walach, 2001), differential placebo effects imply that the medication with the greatest effect compared with its placebo control is not necessarily the most effective. This emphasizes the importance of direct head‐to‐head comparisons to find the medication and route of administration that is most effective for a specific symptom or disease. Furthermore, the differential results for pain and itch indicate that research showing placebo effects on pain cannot directly be generalized to other symptoms, even when underlying mechanisms largely overlap, as with itch. In clinical practice, it is important to take patients’ and doctors’ expectations into account. Keeping in mind the influence of the information a physician provides when administering medication on expectations and consequently treatment outcome (Peerdeman et al., 2016a), the effectiveness of medication, as well as treatment adherence, might be enhanced by actively discussing a patients’ expectations about the available or preferred route of administration. For example, when a physician prescribes topical medication for pain, relatively low effectiveness expectations can be enhanced by expressing the intended positive outcomes and might possibly also be enhanced by highlighting associated characteristics of the route such as safety and ease of use. Alternatively, on some occasions when several equally effective routes are available, it may be possible to select the route the patient expects to be most effective for administering a particular medication or to switch to a different route if a patient's previous experiences were negative (Hofmann et al., 2014).

In conclusion, we found that the expected effectiveness of medication depended on both the route of administration (oral, injection or topical) and the targeted symptom (pain or itch). In addition, the expected effectiveness was found to be associated with expectations about other characteristics of the routes (onset, duration, safety and ease of use). Most importantly, our results indicate that findings from pain research cannot readily be translated into other symptoms. Instead, the findings suggest that differential placebo effects exist, and multiple factors, not merely invasiveness of the route of administration, are at play. The current findings have important implications for the interpretation of placebo‐controlled trials and suggest that medication effects may be enhanced when taking the route of administration into account in clinical practice.

## Author contributions

All authors contributed to the conception and design of the study. K.J.P. & J.T. collected and analysed the data. All authors contributed to the interpretation of the results. All authors contributed to the manuscript for important intellectual content and approved the manuscript.

## Supporting information


**Appendix S1.** Questionnaire Expectations about Medication (English translation).
**Table S1.** Confirmatory principal components analysis of the expected effectiveness of the six forms of medication administration for relieving pain.
**Table S2.** Confirmatory principal components analysis of the expected effectiveness of the six forms of medication administration for relieving itch.
**Table S3.** Means (± standard deviations) of expected effectiveness of medication and expectations about other characteristics of the routes, as rated on 0–100 visual analogue scales.
**Table S4.** Comparisons of expected effectiveness of medication and of expectations about other characteristics of the routes.
**Table S5.** Associations of the expected effectiveness of medication with expectations about other characteristics of the routes and with respondent characteristics, both across routes of administration and symptoms (*overall*) and separately per route of administration and symptom.
