# Supplementary material for: Expectations about the effectiveness of pain‐ and itch‐relieving medication administered via different routes
Source: Eur J Pain. 2017 Dec 20;22(4):774–83. doi: 10.1002/ejp.1163 (PMC5873387; doi:10.1002/ejp.1163)
Supplement: Supplementary file 1 — Appendix S1. Questionnaire Expectations about Medication (English translation). Table S1. Confirmatory principal components analysis of the expected effectiveness of the six forms of medication administration for relieving pain. Table S2. Confirmatory principal components analysis of the expected effectiveness of the six forms of medication administration for relieving itch. Table S3. Means (± standard deviations) of expected effectiveness of medication and expectations about other characteristics of the routes, as rated on 0–100 visual analogue scales. Table S4. Comparisons of expected effectiveness of medication and of expectations about other characteristics of the routes. Table S5. Associations of the expected effectiveness of medication with expectations about other characteristics of the routes and with respondent characteristics, both across routes of administration and symptoms (overall) and separately per route of administration and symptom. [file EJP-22-774-s001.docx]

**Supporting information**

**Appendix S1:** Questionnaire Expectations about Medication (English translation).

**Questions on expectations about medication**

In this questionnaire, you will be asked what you expect about different forms of medication, such as a pill or cream.

You can answer each question on a scale of *not at all* to *very much.* You can see an example of this scale below. Answer by moving the bar to a location that fits best with your opinion. You can do so by clicking on the bar and dragging it to the desired location on the scale. You can also directly click on the desired location.

At each question, the bar is automatically located in the middle of the scale. If this position matches with your answer, could you then still click on the bar? Otherwise, the software will consider the question as unanswered.

There are no right or wrong answers. We are interested in your personal opinion.

|  |  | |  | |  | |  |  |  | |  | |  | |  |  |  |
| --- | --- | --- | --- | --- | --- | --- | --- | --- | --- | --- | --- | --- | --- | --- | --- | --- | --- |
|  |  | |  | |  | |  |  |  | |  | |  | |  |  |  |
|  |  | |  | |  | |  |  |  | |  | |  | |  |  |  |
| Not at all | | |  | |  | |  | | | |  | |  | | Very much | | |

**Description of the forms of medication**

The questionnaire is about 6 forms of medication. Below you can find a brief description of each form.

[presented in random order]

| 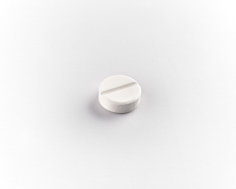 | **Tablet**  A tablet is a round, flat pill. The medication is contained as a powder in this pill. A tablet is taken via the mouth. |
| --- | --- |
| 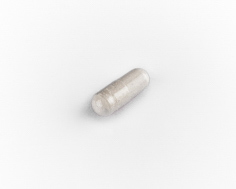 | **Capsule**  A capsule is an oval container. The medication is contained as a powder in this container. A capsule is taken via the mouth. |
| 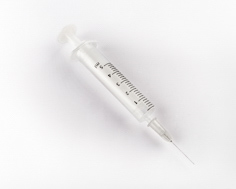 | **Syringe**  A syringe is a vial with a hollow needle attached to it. The medication is dissolved in a fluid and is contained in the vial. The fluid is injected into the body via the needle. |
| 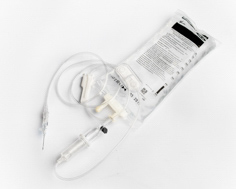 | **Infusion**  An infusion consists of a small bag that is connected to a hollow needle via a tube. The medication is dissolved in a fluid and is contained in the bag. The fluid enters the body via the needle. |
| 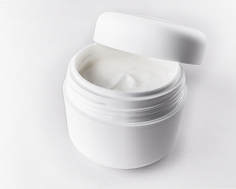 | **Cream**  Cream is a fatty spreadable substance. The medication is dissolved in this substance. Cream is applied to the skin. |
| 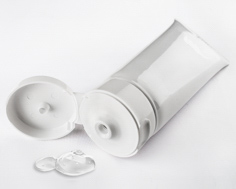 | **Gel**  Gel is a transparent spreadable substance. The medication is dissolved in this substance. Gel is applied to the skin. |

**Effective against pain/itch**

How effective do you think **[pain-relieving/itch-relieving] medications** are when they are used in the following forms?

- tablet
- capsule
- syringe
- infusion
- cream
- gel

|  |  | |  | |  | |  |  |  | |  | |  | |  |  |  |
| --- | --- | --- | --- | --- | --- | --- | --- | --- | --- | --- | --- | --- | --- | --- | --- | --- | --- |
|  |  | |  | |  | |  |  |  | |  | |  | |  |  |  |
|  |  | |  | |  | |  |  |  | |  | |  | |  |  |  |
| Not effective at all | | |  | |  | |  | | | |  | |  | | Very much effective | | |

[Symptoms and forms (always accompanied by photo) are presented in random order.]

**General characteristics**

To what extent do you think each of the following characteristics applies to a [**form**]?

- side effects
- long-lasting effect
- rapid onset
- safe
- frightening
- expensive
- easy to use

|  |  | |  | |  | |  |  |  | |  | |  | |  |  |  |
| --- | --- | --- | --- | --- | --- | --- | --- | --- | --- | --- | --- | --- | --- | --- | --- | --- | --- |
|  |  | |  | |  | |  |  |  | |  | |  | |  |  |  |
|  |  | |  | |  | |  |  |  | |  | |  | |  |  |  |
| Not applicable at all | | |  | |  | |  | | | |  | |  | | Very much applicable | | |

[Forms (always accompanied by photo) and characteristics are presented in random order.]

**Table S1.** Confirmatory principal components analysis of the expected effectiveness of the six forms of medication administration for relieving pain.

|  | Component | | |
| --- | --- | --- | --- |
|  | 1 | 2 | 3 |
| Tablet | -0.06 | 0.02 | **-0.96** |
| Capsule | 0.12 | -0.02 | **-0.87** |
| Syringe | **0.91** | -0.01 | -0.04 |
| Infusion | **0.93** | 0.02 | 0.01 |
| Cream | -0.08 | **0.92** | -0.11 |
| Gel | 0.08 | **0.96** | 0.09 |

*Note*. Pattern matrix of the oblimin rotation with Kaiser normalization.

**Table S2.** Confirmatory principal components analysis of the expected effectiveness of the six forms of medication administration for relieving itch.

|  | Component | | |
| --- | --- | --- | --- |
|  | 1 | 2 | 3 |
| Tablet | -0.06 | 0.01 | **-1.00** |
| Capsule | 0.09 | -0.02 | **-0.90** |
| Syringe | **0.92** | -0.02 | -0.03 |
| Infusion | **0.97** | 0.02 | 0.02 |
| Cream | ≈-0.01 | **0.95** | 0.02 |
| Gel | <0.01 | **0.95** | -0.02 |

*Note*. Pattern matrix of the oblimin rotation with Kaiser normalization.

**Table S3.** Means (± standard deviations) of expected effectiveness of medication and expectations about other characteristics of the routes, as rated on 0-100 visual analogue scales.

| Characteristics | Overall | |  | Oral | | Injection | | Topical | |
| --- | --- | --- | --- | --- | --- | --- | --- | --- | --- |
| Effectiveness |  |  |  |  |  |  |  |  |  |
| Pain relief | 67.9 | *(13.4)* |  | 73.0 | *(15.6)* | 81.8 | *(16.8)* | 48.8 | *(22.0)* |
| Itch relief | 63.0 | *(15.4)* |  | 58.3 | *(21.7)* | 60.9 | *(24.6)* | 69.9 | *(19.4)* |
|  |  |  |  |  |  |  |  |  |  |
| Side effects | 48.2 | *(12.6)* |  | 53.7 | *(15.8)* | 51.6 | *(16.3)* | 39.3 | *(17.6)* |
| Long-lasting effect | 57.4 | *(12.0)* |  | 59.0 | *(14.8)* | 66.3 | *(16.2)* | 46.8 | *(18.2)* |
| Rapid onset | 64.5 | *(10.7)* |  | 61.0 | *(14.7)* | 77.9 | *(15.6)* | 54.4 | *(18.1)* |
| Safe | 64.0 | *(12.8)* |  | 63.8 | *(16.3)* | 58.8 | *(17.8)* | 69.3 | *(15.9)* |
| Frightening | 32.2 | *(16.1)* |  | 26.1 | *(19.7)* | 52.7 | *(26.9)* | 17.9 | *(18.4)* |
| Expensive | 56.9 | *(12.5)* |  | 50.7 | *(16.8)* | 69.7 | *(16.3)* | 50.2 | *(17.6)* |
| Easy to use | 65.0 | *(12.7)* |  | 76.8 | *(16.7)* | 38.6 | *(25.3)* | 79.7 | *(16.5)* |

**Table S4.** Comparisons of expected effectiveness of medication and of expectations about other characteristics of the routes.

| Characteristics | Main effect route of administration |  | Oral vs Injection | Oral vs Topical | Injection vs Topical |
| --- | --- | --- | --- | --- | --- |
| Effectiveness |  |  |  |  |  |
| Pain relief | *F* (1.66, 839.37) = 628.29, *p* < .001, *η_G_²* = **.47** ^a^ |  | *F* (1, 507) = 148.61, *p* < .001, *η_G_²* = **.11** ^b^ | *F* (1, 507) = 572.39, *p* < .001, *η_G_²* = **.37** ^b^ | *F* (1, 507) = 875.34, *p* < .001*, η_G_²* = **.50** ^b^ |
| Itch relief | *F* (1.50, 761.86) = 50.12, *p* < .001,  *η_G_²* = **.07** ^a^ |  | *F* (1, 507) = 10.25, *p* = .006, *η_G_²* = .01 *^b^* | *F* (1, 507) = 80.28, *p* < .001*, η_G_²* = **.10** *^b^* | *F* (1, 507) = 38.58, *p* < .001, *η_G_²* = .05 ^b^ |
|  |  |  |  |  |  |
| Side effects ^c^ | *F* (1.88, 953.10) = 178.58, *p* < .001, *η_G_^2^* = **.19** |  | *F* (1, 507) = 9.17, *p* = .003, *η_G_^2^* = .01 | *F* (1, 507) = 275.16, *p* < .001, *η_G_^2^* = **.22** | *F* (1, 507) = 194.10, *p* < .001, *η_G_^2^* = **.16** |
| Long-lasting effect ^c^ | *F* (1.74, 880.62) = 260.79, *p* < .001, *η_G_^2^* = **.27** |  | *F* (1, 507) = 93.01, *p* < .001, *η_G_^2^* = **.08** | *F* (1, 507) = 240.05, *p* < .001, *η_G_^2^* = **.17** | *F* (1, 507) = 367.04, *p* < .001, *η_G_^2^* = **.31** |
| Rapid onset ^c^ | *F* (1.84, 934.86) = 337.31, *p* < .001, *η_G_^2^* = **.35** |  | *F* (1, 507) = 421.48, *p* < .001, *η_G_^2^* = **.31** | *F* (1, 507) = 53.33, *p* < .001, *η_G_^2^* = .05 | *F* (1, 507) = 494.71, *p* < .001, *η_G_^2^* = **.39** |
| Safe ^c^ | *F* (1.88, 951.07) = 82.37, *p* < .001,  *η_G_^2^* = **.10** |  | *F* (1, 507) = 36.71, *p* < .001, *η_G_^2^* = .03 | *F* (1, 507) = 59.17, *p* < .001, *η_G_^2^* = .04 | *F* (1, 507) = 135.01, *p* < .001, *η_G_^2^* = **.12** |
| Frightening ^c^ | *F* (1.47, 743.07) = 499.74, *p* < .001, *η_G_^2^* = **.42** |  | *F* (1, 507) = 437.48, *p* < .001, *η_G_^2^* = **.32** | *F* (1, 507) = 125.33, *p* < .001, *η_G_^2^* = **.07** | *F* (1, 507) = 664.77, *p* < .001, *η_G_^2^* = **.44** |
| Expensive ^c^ | *F* (1.81, 919.59) = 319.81, *p* < .001, *η_G_^2^* = **.31** |  | *F* (1, 507) = 443.51, *p* < .001, *η_G_^2^* = **.32** | *F* (1, 507) = 0.41, *p* = .53, *η_G_^2^* < .01 | *F* (1, 507) = 392.69, *p* < .001, *η_G_^2^* = **.32** |
| Easy to use ^c^ | *F* (1.57, 795.84) = 751.18, *p* < .001, *η_G_^2^* = **.55** |  | *F* (1, 507) = 829.36, *p* < .001, *η_G_^2^* = **.52** | *F* (1, 507) = 12.75, *p* < .001, *η_G_^2^* = .01 | *F* (1, 507) = 948.99, *p* < .001, *η_G_^2^* = **.55** |

*Note*. ^a^ *p* values of these repeated measures analysis of variance are corrected according to inheritance procedure (*p* / ⅗ [13]); ^b^ *p* values of these repeated measures analysis of variance are corrected according to inheritance procedure (*p* x 4 [13]); ^c^ *p* values are unadjusted. Medium and large effect sizes are printed in bold.

**Table S5.** Associations of the expected effectiveness of medication with expectations about other characteristics of the routes and with respondent characteristics, both across routes of administration and symptoms (*overall*) and separately per route of administration and symptom.

|  |  | Expected effectiveness | | | | | | | | | |
| --- | --- | --- | --- | --- | --- | --- | --- | --- | --- | --- | --- |
|  |  | Overall |  | Pain | | |  | Itch | | |  |
|  | ^a^ |  |  | Oral | Injection | Topical |  | Oral | Injection | Topical |  |
| **Other characteristics of the routes** ^b^ |  |  |  |  |  |  |  |  |  |  |  |
| Side effects | *r* | -.06 |  | -.10^*^ | -.03 | .05 |  | -.03 | -.02 | <.01 |  |
| Long-lasting effect | *r* | **.44^***^** |  | **.42^***^** | **.36^***^** | **.39^***^** |  | **.31^***^** | .26^***^ | .26^***^ |  |
| Rapid onset | *r* | **.49^***^** |  | **.43^***^** | **.57^***^** | **.36^***^** |  | .24^***^ | .25^***^ | **.37^***^** |  |
| Safe | *r* | **.42^***^** |  | **.37^***^** | .27^***^ | .17^***^ |  | .18^***^ | .26^***^ | .26^***^ |  |
| Frightening | *r* | -.20^***^ |  | -.23^***^ | -.05 | .05 |  | -.11^*^ | -.19^***^ | -.20^***^ |  |
| Expensive | *r* | .05 |  | -.03 | .23^***^ | -.03 |  | .06 | .03 | -.06 |  |
| Easy to use | *r* | **.31^***^** |  | **.31^***^** | .02 | .04 |  | .12^**^ | .23^***^ | .27^***^ |  |
|  |  |  |  |  |  |  |  |  |  |  |  |
| **Demographics** |  |  |  |  |  |  |  |  |  |  |  |
| Age | *r* | .04 |  | -.01 | -.02 | .01 |  | .07 | .10* | -.05 |  |
| Sex | *η_G_^2^* | < .01 |  | < .01 | < .01 | < .01 |  | < .01 | < .01 | .01* |  |
| Educational level | *η_G_^2^* | < .01 |  | < .01 | < .01 | < .01 |  | .02* | .02* | .01 |  |
| Religious or ideological affiliation | *η_G_^2^* | < .01 |  | < .01 | < .01 | < .01 |  | < .01 | < .01 | < .01 |  |
| Marital status | *η_G_^2^* | < .01 |  | < .01 | < .01 | < .01 |  | < .01 | < .01 | < .01 |  |
|  |  |  |  |  |  |  |  |  |  |  |  |
| **Health** |  |  |  |  |  |  |  |  |  |  |  |
| Currently in treatment for long-lasting medical or psychological complaints or diseases | *η_G_^2^* | < .01 |  | < .01 | < .01 | < .01 |  | .01 | < .01 | < .01 |  |
| Chronic pain past | *η_G_^2^* | .01* |  | < .01 | .01 | < .01 |  | - | - | - |  |
| Chronic itch past | *η_G_^2^* | < .01 |  | - | - | - |  | < .01 | < .01 | < .01 |  |
| Chronic pain present | *η_G_^2^* | .01 |  | < .01 | .01 | < .01 |  | - | - | - |  |
| Chronic itch present | *η_G_^2^* | < .01 |  | - | - | - |  | < .01 | < .01 | < .01 |  |
| Current pain intensity | *r* | .05 |  | .04 | .07 | -.04 |  | - | - | - |  |
| Current itch intensity | *r* | -.01 |  | - | - | - |  | .01 | -.02 | -.01 |  |
| Physical health status  (SF-12) | *r* | -.06 |  | -.01 | -.07 | .07 |  | -.13** | -.11* | .04 |  |
| Mental health status  (SF-12) | *r* | -.02 |  | -.01 | -.04 | .04 |  | -.01 | -.01 | -.06 |  |
|  |  |  |  |  |  |  |  |  |  |  |  |
| **Frequency of medication use** ^b^ |  |  |  |  |  |  |  |  |  |  |  |
| Frequency of pain-relieving medication use | *r* | .09 |  | .05 | .10^*^ | .10^*^ |  | - | - | - |  |
| Frequency of itch-relieving medication use | *r* | .11* |  | - | - | - |  | .19^***^ | .02 | .15^**^ |  |
|  |  |  |  |  |  |  |  |  |  |  |  |

| Medication attitude |  |  |  |  |  |  |  |  |  |  |
| --- | --- | --- | --- | --- | --- | --- | --- | --- | --- | --- |
| Beliefs about medication - general harm (BMQ) | *r* | -.16^***^ |  | -.24^***^ | -.16^***^ | -.01 |  | -.14^**^ | -.03 | -.11^*^ |
| Beliefs about medication - general overuse (BMQ) | *r* | -.11^*^ |  | -.15^**^ | -.05 | -.04 |  | -.09^*^ | -.06 | -.04 |
| Health care employee (past or present) | *η_G_^2^* | .02** |  | < .01 | .01** | < .01 |  | .02** | .02** | < .01 |
| If health care employee (*n* = 76): prescribed medication (past or present) | *η_G_^2^* | .05 |  | .01 | .03 | **.11**** |  | .03 | .01 | .04 |
|  |  |  |  |  |  |  |  |  |  |  |
| **Personality characteristics** |  |  |  |  |  |  |  |  |  |  |
| Optimism (LOT-R) | *r* | .08 |  | .09 | .09^*^ | .07 |  | .01 | -.02 | .11^*^ |
| Neuroticism (EPQ-RSS) | *r* | -.01 |  | < .01 | < .01 | -.02 |  | -.02 | -.03 | .04 |

*Note*. ^a^ *r* = Pearson correlation coefficient (for continuous variables); *η_G_^2^* = generalized eta squared (for categorical variables). *^b^* correlations between corresponding routes are reported (i.e., oral *vs.* oral; injection *vs.* injection, and topical *vs.* topical)*.* * *p* < .05, ** *p* < .01, *** *p* < .001; *p* values are unadjusted. Medium and large effect sizes are printed in bold.
